# Effect of Green Synthesized ZnO-NPs on Growth, Antioxidant System Response and Bioactive Compound Accumulation in *Echinops macrochaetus*, a Potential Medicinal Plant, and Assessment of Genome Size (2C DNA Content)

**DOI:** 10.3390/plants12081669

**Published:** 2023-04-17

**Authors:** Salim Khan, Fahad Al-Qurainy, Abdulrahman Al-hashimi, Mohammad Nadeem, Mohamed Tarroum, Hassan O. Shaikhaldein, Abdalrhaman M. Salih

**Affiliations:** Department of Botany and Microbiology, College of Science, King Saud University, P.O. Box 2455, Riyadh 11451, Saudi Arabia

**Keywords:** nano-fertilizers, genome size, medicinal plants, polyphenolic compounds, antioxidant system response

## Abstract

*Echinops macrochaetus* is a medicinal plant that can be used to cure various diseases. In the present study, plant-mediated zinc oxide nanoparticles (ZnO-NPs) were synthesized using an aqueous leaf extract of the medicinal plant *Heliotropium bacciferum* and characterized using various techniques. *E. macrochaetus* was collected from the wild and identified using the internal transcribed spacer sequence of nrDNA (ITS-nrDNA), which showed the closeness to its related genus in a phylogenetic tree. The effect of synthesized biogenic ZnO-NPs was studied on *E. macrochaetus* in a growth chamber for growth, bioactive compound enhancement and antioxidant system response. The irrigation of plants at a low concentration of ZnO-NPs (T1 = 10 mg/L) induced more growth in terms of biomass, chlorophyll content (273.11 µg/g FW) and carotenoid content (135.61 µg/g FW) than the control and other treatments (T2-20 mg/L and T3-40 mg/L). However, the application of a high concentration of ZnO-NPs (20 and 40 mg/L) increased the level of antioxidant enzymes (SOD, APX and GR), total crude and soluble protein, proline and TBARS contents. The accumulations of the compounds quercetin-3-β-D-glucoside, luteolin 7-rutinoside and p-coumaric acid were greater in the leaf compared to the shoot and root. A minor variation was observed in genome size in treated plants as compared to the control group. Overall, this study revealed the stimulatory effect of phytomediated ZnO-NPs, which act as bio-stimulants/nano-fertilizers as revealed by more biomass and the higher production of phytochemical compounds in different parts of the *E. macrochaetus*.

## 1. Introduction

The genus *Echinops* belongs to the family Asteraceae, which comprises 1600–1700 genera and 24,000–30,000 species [1]. Many species of *Echinops* are traditionally employed to treat various diseases, mainly in Africa and Asia. Different types of secondary metabolites, such as flavonoids, phenolics, alkanoids and essential oils, are found in different parts of plant of genus *Echinops*, due to which it has been used in traditional medicine to treat various diseases [2] (review). The key bioactive constituents of the genus *Echinops* are thiophenes, which are biosynthetically derived from fatty acids and reduced sulfur [3]. The root of the plant is the important source of the thiophenes, while most of the flavonoids and terpenes were isolated from the aerial part/the whole plant. *Echinops macrochaetus* is a medicinal plant that is found in a few places in Saudi Arabia. *E. macrochaetus* showed cytotoxic activity due to the presence of Macrochaetosides A, Macrochaetosides B (sesquiterpene glycosides) and cyclostenol [4]. Different compounds were isolated from the roots of *E. macrochaetus* including 5-(but-3-en-1-ynyl)-2,2′-bithiophene, 5-(penta-1,3-diynyl)-2-(3-chloro-4-hydoxy-but-1-ynyl)-thiophene and stigmasterol [4,5].

In higher plants, the flavonoid biosynthetic pathway has been well defined, and several key enzymes are involved in this pathway [6,7]. Regardless of their structural differences and physiological functions, these metabolites are controlled by complex biosynthetic pathways, sharing the first three conversion steps [8]. In this way, the boundary between primary and secondary metabolism is coordinated by particular metabolites (cinnamic acid and p-coumaric acid) [8]. Among many secondary metabolites, phenolic compounds have numerous valuable effects on humans to cure various diseases, such as cancer and neurodegenerative disorders [9,10]. Many flavonoids, such as luteolin (*Echinops niveus*) [11] and rutin (*Echinops heterophyllus*) [12], and phenolic compounds, including coumarins, phenylpropanoids, and lignans, were reported [13,14,15]. Many phenolic acids have been reported, such as *p*-coumaric acid (4-hydroxycinnamic serves as a precursor of other phenolic compounds and is found in many plant species) which exhibits various bioactivities, including antioxidant, anti-inflammatory, antimutagenic, anti-ulcer, antiplatelet and anti-cancer activities, in addition to mitigating oxidative cardiac damage, atherosclerosis, neuronal injury, UV-induced damage to ocular tissues, anxiety, diabetes and gout [16].

Zinc is an essential micronutrient and plays important functions in different enzyme activities. Zinc (Zn) and potassium (K) are essential elements in plant growth and metabolism and play vital parts in salt stress tolerance [17]. The bio-accessibility of Zn in soils is often poor, as it binds to insoluble compounds. This issue can be resolved by using fertilizers in different forms, such as chelates, complexes and salts. A viable substitute may be nano-fertilizers containing Zn in the form of zinc oxide nanoparticles (ZnO-NPs) that may be more efficiently absorbed by plants [18]. ZnO-NPs are considered the most significant of the metal oxide NPs due to their distinctive physical and chemical properties, which enhance their applicability aspects [19]. A rising body of data suggests that the positive effects of given molecules are usually amplified when these are used in the form of nanoparticles (NPs) [20]. Synthesized biogenic ZnO-NPs have many applications, such as in photodegradation, antibacterial and antifungal uses, and in treating hyperthermia [21,22]. Many studies have highlighted the potential use of nanotechnology in crop production by improving nutritional yield and nutraceutical value [23]. Nanoparticles are important abiotic elicitors employed for obtaining improved yields of plant-derived medicinal compounds. The effects of NPs mainly depend on their type, concentration, size, duration of exposure, method and even on the species type exposed to the nanoparticles [24]. Usually, NPs have divergent physico-chemical properties that facilitate them to inter-relate with plant metabolic systems in a beneficial way. The application of a high concentration of nanoparticles causes oxidative stress, and owing to harsh conditions, elicits and aggregates cell death symptoms. This is produced by the accumulation of reactive oxygen species (ROS) in the cell. ROS accumulation has been linked with many undesirable effects, including lipid peroxidation (TBARS content), electrolyte leakage and membrane damage. From this perspective, advancing plant capacity to detoxify ROS is a viable means of lifting plant performance. ROS are detoxified by the antioxidant defense elements, involving both enzymes and metabolites [25].

In the past few years, ZnO-NPs have received great attention due their ability to improve nutrient accumulation by plants for enhancing the growth, biomass and quality of crops [26,27]. In addition, some reports have described positive aspects of ZnO-NPs regarding their impacts on plant growth, antioxidant system response [28], secondary metabolite production [29] and induction of photosynthetic efficiency [30]. However, high concentrations of ZnO-NPs can be phytotoxic, genotoxic [31] and limit the plant growth and yield. Therefore, the application of an optimum concentration of nanoparticles is necessary to protect the environment as well as the biotic system. *Echinops macrochaetus* is an important medicinal plant, as reported in the literature, and is endemic to Saudi Arabia. Since, the population number of *E. macrochaetus* is small, more attention must be paid to its propagation using nanobiotechnology approaches. In the past, no study has been reported on *E. macrochaetus* related to the application of biogenic nanoparticles for the enhancement of biomass and bioactive compounds. Therefore, it is necessary to study the effect of biogenic ZnO-NPs on biomass, morphometric traits and polyphenolic compound accumulation in *E. macrochaetus*. Further, the impact of synthesized biogenic nanoparticles was assessed on biochemical parameters and genome size (2C DNA content) in *E. macrochaetus* at different treatment doses of ZnO-NP application.

## 2. Results

**Figure 1:**
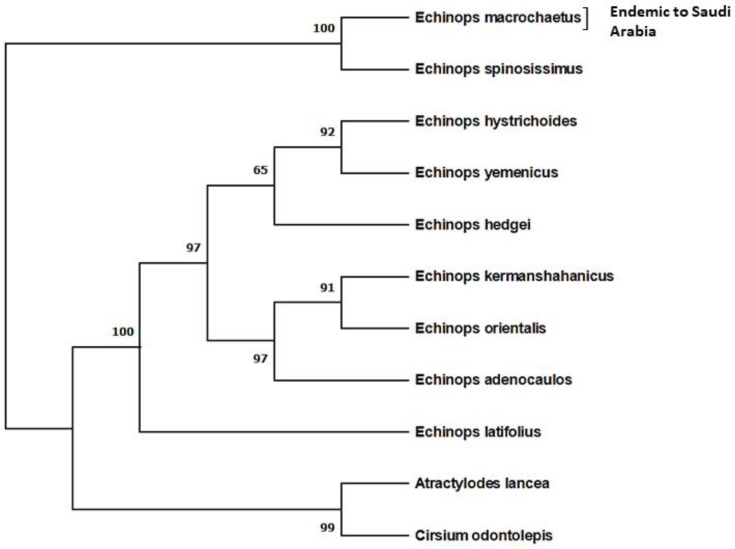
Phylogenetic reconstruction of *Echinops macrochaetus* with other species of *Echinops* using Maximum Parsimony method.

The collected plant, *Echinops macrochaetus*, was identified by a taxonomist as well as molecular marker before the application of biogenic ZnO-NPs. The internal transcribed spacer sequence of nrDNA (nrDNA-ITS) was amplified and sequenced, and a phylogenetic tree was reconstructed for *E. macrochaetus* identification and its relationship to other species of *Echinops* available in the GenBank database (https://www.ncbi.nlm.nih.gov/ (accessed on 16 December 2022)) using MEGA X software version 11 [32] (Figure 1). The nrDNA-ITS sequence of *E. macrochaetus* was submitted to the GenBank database (accession No: OQ540950). The Maximum Parsimony (MP) method was used for the reconstruction of phylogenetic tree. The percentage of replicate trees in which the related taxa clustered together in the bootstrap test (1000 replicates) are shown next to the branches [33]. The plant species *Atractylodes lancea* (KF301177) and *Cirsium odontolepis* (MN918951), downloaded from the GenBank database, were used as the outgroup, as shown in Figure 1. *E. macrochaetus* clustered and showed more closeness with *E. spinosissimus* (accession no. HE687348 downloaded from GenBank: https://www.ncbi.nlm.nih.gov/ (accessed on 16 December 2022) than the other species of *Echinops*, including *Echinops hystrichoides* (GU116515), *Echinops hedgei* (AY538648), *Echinops latifolius* (MH711533), *Echinops kermanshahanicus* (KT819534), *Echinops yemenicus* (GU116548), *Echinops adenocaulos* (MW621242) and *Echinops orientalis* (MW621260), download from NCBI (https://www.ncbi.nlm.nih.gov/ (accessed on 16 December 2022)). Thus, the nrDNA-ITS marker used in this study confirmed the identification of *E. macrochaetus* and the phylogenetic relationship to other species of this genus.

### 2.1. Biosynthesis and Characterization of ZnO-NPs

**Figure 2:**
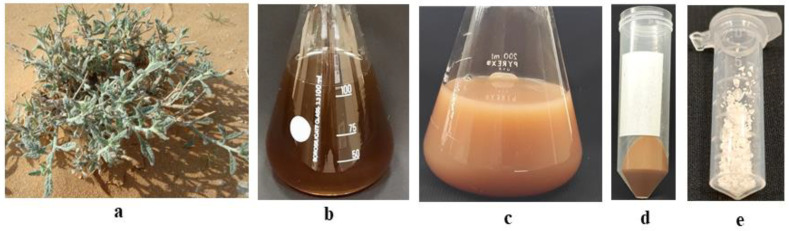
Biosynthesis of ZnO-NPs using the aqueous leaf extract of *Heliotropium bacciferum*: (**a**) *H*. *bacciferum* in natural habitat; (**b**) aqueous leaf extract; (**c**) synthesized ZnO-NPs; (**d**) ZnO-NPs in pelleted form with secondary metabolites; (**e**) powder form of synthesized ZnO-NPs.

**Figure 3:**
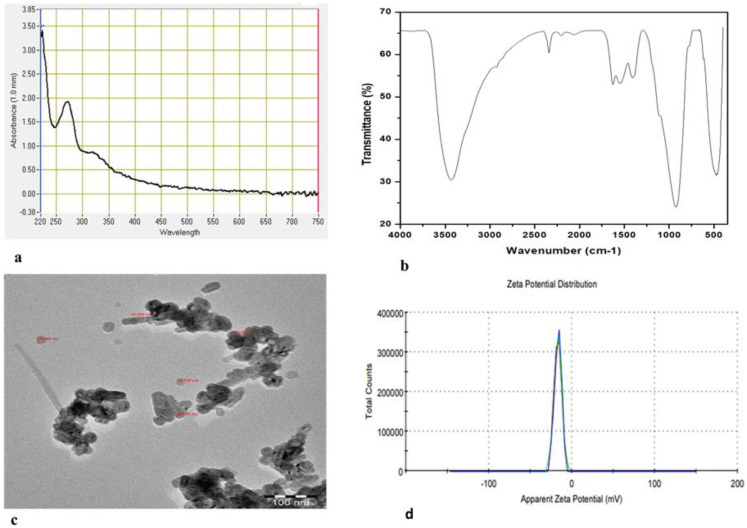
Characterization of synthesized phytomediated ZnO-NPs: (**a**) UV-visible spectrum; (**b**) Fourier transmission infrared (FTIR); (**c**) transmission electron microscopy (TEM); (**d**) zeta potential.

The fabrication of ZnO-NPs using an aqueous extract of *Heliotropium bacciferum* (family Boraginaceae) was performed (Figure 2) and described using different techniques (Figure 3). The medicinal plant *H*. *bacciferum* was identified using the internal transcribed spacer sequence of nrDNA (GenBank accession no. KF805115). The synthesized ZnO-NPs were characterized using UV–Vis spectroscopy, Fourier transform infrared (FT-IR) spectroscopy and transmission electron microscopy (TEM) (Figure 3). The synthesis of ZnO-NPs started as the 0.1 N NaOH was added in the suspension of plant extract and Zn (NO_3_)_2_. The sharp peak of the UV spectra at 276 nm showed the pure synthesis of ZnO-NPs. The plant-mediated synthesis of ZnO-NPs checked in the range of 220–750 nm using a UV–Vis spectrophotometer (NanoDrop 8000 spectrophotometer Wilmington, NC, USA). FTIR spectra were obtained in the range of 400–4000 cm^−1^. The absorption bands detected by FTIR at different wavelengths were 3432.70 cm^−1^, 2342.20 cm^−1^, 1627.28 cm^−1^, 1547.49 cm^−1^, 1406.72 cm^−1^, 922.98 cm^−1^ and 474.92 cm^−1^, which are associated with diverse functional groups available in the plant extract of *H. bacciferum*. The absorption band detected at 3432.70 cm^−1^ revealed a broad and intense peak that resulted from stretching and vibration of an O-H functional group. The peak at 1627 cm^−1^ was assigned to a compound having a functional group (C=C stretching). Similarly, the band at 1406.72 cm^−1^ is associated with a functional group and O-H bending. Thus, the synthesized phytomediated ZnO-NPs confirm the presence of various functional groups in the leaf extract of *H. bacciferum* that are responsible for the lessening and stabilization of ZnO-NPs. The average zeta potential value of ZnO-NPs was detected (−17 mV) with a conductivity 0.11 mS/cm, which shows its stability. The transmission electron microscopy (TEM) of ZnO-NPs was detected in the range of 18.730–25.531 nm, which indicates the synthesis of nanoparticles. Thus, the characterization of synthesized biogenic ZnO-NPs confirmed their application on *E. macrochaetus* for more biomass and bioactive compound production.

#### 2.1.1. Effect of ZnO-NPs on Morphological Traits

**Figure 4:**
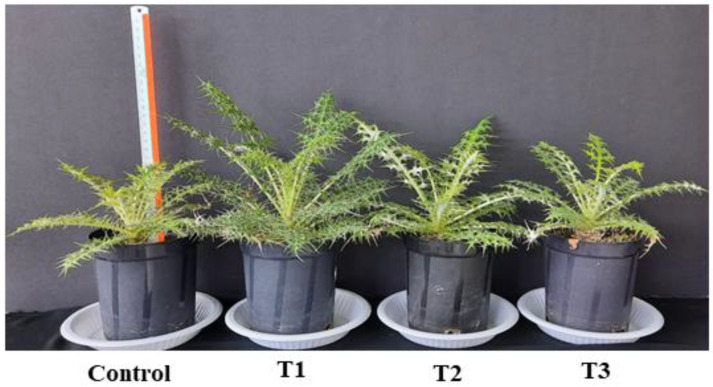
Effect of ZnO-NPs on morphological traits of *E. macrochaetus*. ZnO-NP treatments: control (0 mg/L); T1 (10 mg/L); T2 (20 mg/L) and T3 (40 mg/L).

Four-week-old *E. macrochaetus* plants grown in pots were irrigated with different concentrations of ZnO-NPs (100 mL each pot) (C = 0 mg/L, T1 = 10 mg/L, T2 = 20 mg/L and T3 = 40 mg/L per month), and another time with double-distilled water (100 mL per pot) every after fifteen days. The plants treated at different concentrations of ZnO-NPs showed variation in growth and morphological traits (Figure 4).

**Figure 5:**
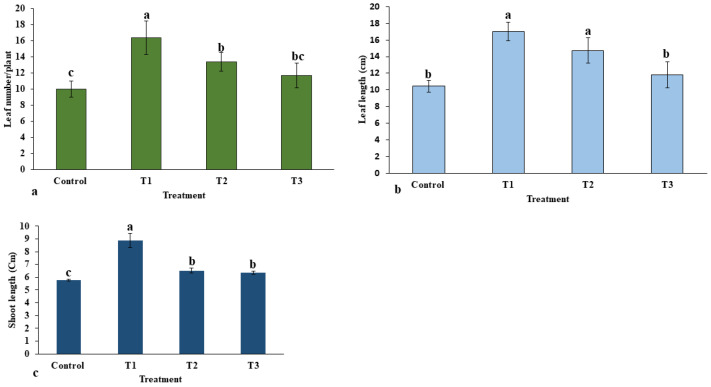
Effect of ZnO-NPs on morphological traits of *E. macrochaetus*: (**a**) leaf number; (**b**) leaf length; (**c**) shoot length. Treatments including control (0 mg/L, ZnO-NPs); T1 (10 mg/L, ZnO-NPs); T2 (20 mg/L, ZnO-NPs) and T3 (40 mg/L, ZnO-NPs). Different letters represent the significant values according to Duncan test at level *p* < 0.05.

The applied various doses of ZnO-NPs on *E. macrochaetus* significantly and non-significantly improved the morphological traits, including leaf number, leaf length and shoot length, compared to the control group. The highest leaf number (16.33 per plant) was recorded in the T1 treatment group (Figure 5a). Among different parts of *E. macrochaetus*, more positive effects of ZnO-NPs were observed on leaf length at 10 and 20 mg/L ZnO-NPs as compared to the control group (Figure 5b). Maximum leaf length (17.03 cm) was recorded in plants treated at 10 mg/L ZnO-NPs. ZnO-NPs (20 and 40 mg/L) showed approximately similar effects on shoot length and leaf number. The effect of ZnO-NPs on shoot length varied significantly and non-significantly among different treatments (Figure 5c). Greater shoot length was recorded in all treatments as compared to the control group, and the highest significant positive effect was observed in treatment T1 (8.87 cm). The results obtained in treatments T2 and T3 on shoot length were found to be non-significant, however, shoot length was increased significantly compared the control group.

#### 2.1.2. Effect of ZnO-NPs on Biomass Accumulation

**Figure 6:**
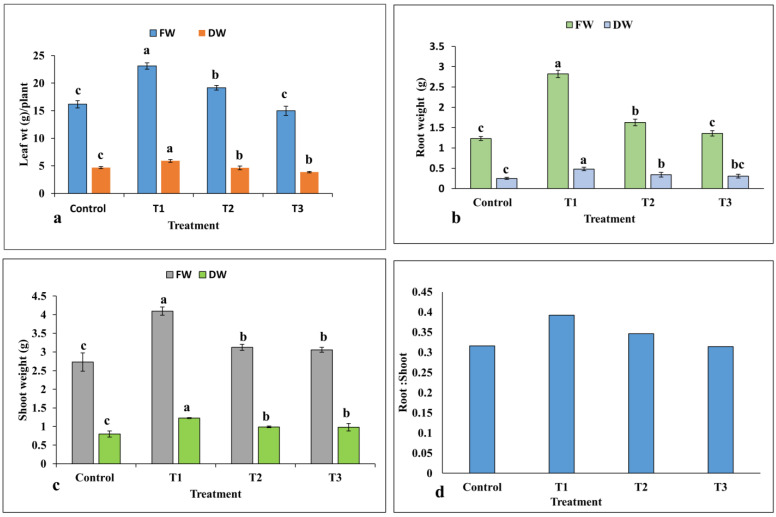
Effects of ZnO-NPs on fresh and dry weights of plant parts: (**a**) leaves; (**b**) roots; (**c**) shoots; (**d**) root to shoot ratio per plant (*n* = 3). Treatments including control (0 mg/L, ZnO-NPs); T1 (10 mg/L, ZnO-NPs); T2 (20 mg/L, ZnO-NPs) and T3 (40 mg/L, ZnO-NPs). Different letters represent the significant values according to the test of Duncan at level *p* < 0.05.

The highest fresh and dry weights of leaves and roots were recorded at the lowest concentration of ZnO-NPs in treatment T1 at 10 mg/L ZnO-NPs compared to the control group (Figure 6a,b). However, the root dry weight was observed to be non-significant between treatments T2 and T3. As with the leaf and root results, shoot fresh and dry weights were increased significantly under different concentrations of ZnO-NPs compared to the control group (Figure 6c). The highest shoot weight (4.09 g per plant) was observed in treatment T1, whereas no significant difference was recorded between treatment T2 and T3. The root and shoot dry weight ratio was found to be the highest in treatment T1, and thereafter it was decreased in all treatments (Figure 6d).

#### 2.1.3. Photosynthetic Pigment Content

**Figure 7:**
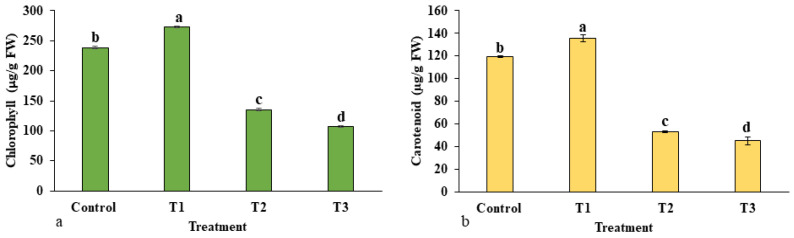
Effects of ZnO-NP on photosynthetic pigments: (**a**) total chlorophyll; (**b**) carotenoid content. Treatments including control (0 mg/L, ZnO-NPs); T1 (10 mg/L, ZnO-NPs); T2 (20 mg/L, ZnO-NPs) and T3 (40 mg/L, ZnO-NPs). Different letters designate a significant difference by Duncan’s multiple range tests at *p* < 0.05.

The total contents of chlorophyll and carotenoids are presented in Figure 7a,b. At different concentrations of ZnO-NPs, both contents were varied. Interestingly, the total chlorophyll and carotenoids sharply increased significantly in treatment T1 (273.11 µg/g FW) with respect to the control group (238.08 µg/g FW), and thereafter their content decreased as the concentration of ZnO-NPs increased in the treatment. Both chlorophyll and carotenoid contents were observed to be low in treatments T2 and T3 as compared to control.

#### 2.1.4. Antioxidant Enzyme Activity

**Figure 8:**
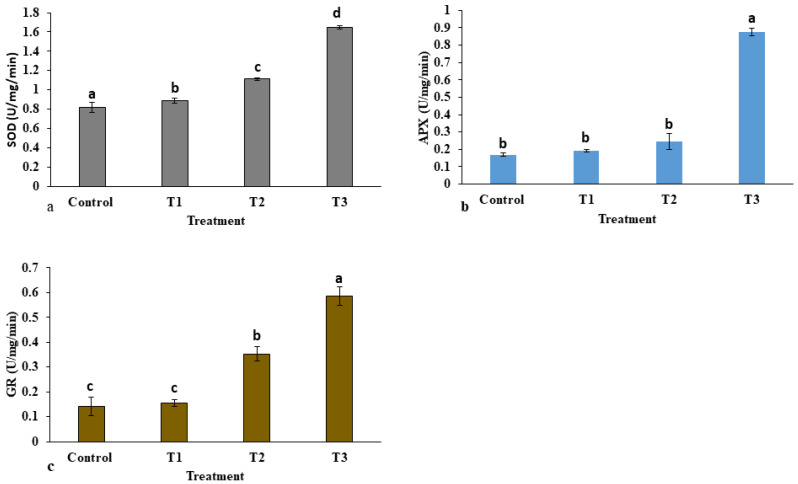
Effect of ZnO-NPs on antioxidant enzyme activities in *E. macrochaetus*: (**a**) superoxide dismutase (SOD); (**b**) ascorbate peroxidase (APX); (**c**) glutathione reductase (GR). Treatments including control (0 mg/L, ZnO-NPs); T1 (10 mg/L, ZnO-NPs); T2 (20 mg/L, ZnO-NPs) and T3 (40 mg/L, ZnO-NPs). Bars represented by different letters indicate significant values according to Duncan’s test (*p* < 0.05).

The activities of antioxidant enzymes in *E. macrochaetus* under different treatment doses of ZnO-NPs are shown in Figure 8. The antioxidant enzyme activities were enhanced at high concentrations of ZnO-NPs, leading to the removal of free radicals from the cell. Compared to the control group, the activity of the SOD enzyme in the leaves under ZnO-NP treatment was increased significantly, and it was observed in a dose-dependent manner. The SOD activity (1.63 U/mg/min) was highest at 40 mg/L ZnO-NPs compared to the activity reported in the control group. However, the activity of the APX enzyme was non-significantly increased in treatments T1 and T2 compared to the control group. There were higher points recorded in APX activity in treatment T3 (0.875 U/mg/min) than in the control group. A non-significant difference was observed in GR activity in treatment T1 compared to control. However, GR activity was significantly increased in treatments T2 and T3 as compared to control.

#### 2.1.5. Estimation of Proline and TBARS Content

**Figure 9:**
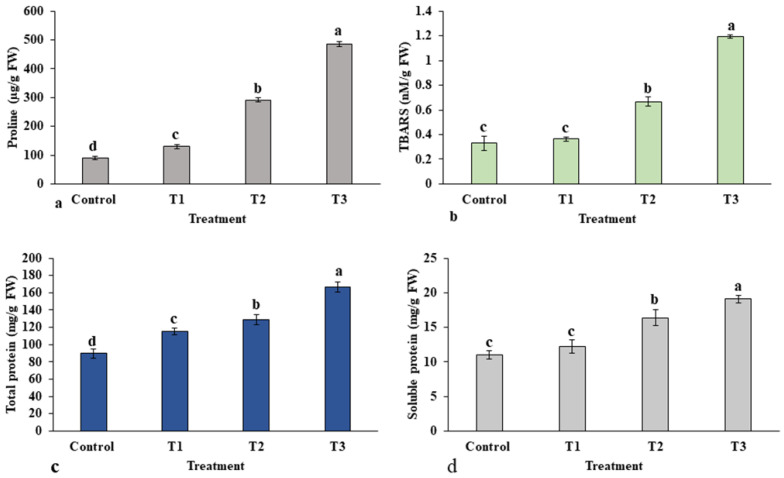
Effect of ZnO-NPs on accumulation of various biochemicals in the leaves of *E. macrochaetus*: (**a**) proline content; (**b**) TBARS content; (**c**) total protein content; (**d**) soluble protein content. Treatments including control (0 mg/L ZnO-NPs); T1 (10 mg/L, ZnO-NPs); T2 (20 mg/L, ZnO-NPs) and T3 (40 mg/L, ZnO-NPs). Bars represented by different letters indicate significant values according to Duncan’s test (*p* < 0.05).

Plants accumulate an array of metabolites, mainly amino acids, when exposed to abiotic stresses. The effect of ZnO-NP treatment on the accumulation of proline is presented in Figure 9a. The content of proline was significantly increased in all treatments in a dose-dependent manner in the leaves of *E. macrochaetus* compared to the control group. A higher accumulation of proline was observed at a high concentration of ZnO-NPs in treatment T3 (485 µg/g FW) compared to the control group (89.77 µg/g FW). The accumulation of TBARS content in treatment T1 was not significant compared to the control group, which confirms the bio-stimulatory effect of a low concentration of ZnO-NPs (10 mg/L). However, treatment T2 (0.66 nM/g FW) and treatment T3 (1.19 nM/g FW) showed significantly higher accumulations of TBARS content compared to control (0.32 nM/g FW) (Figure 9b). Thus, the results show that the 40 mg/L ZnO-NPs concentration induced the accumulation of both proline and TBARS in *E. macrochaetus*.

#### 2.1.6. Total Protein Content

Figure 9c,d show the contents of the total protein and soluble protein extracted from the leaves of *E. macrochaetus*. Interestingly, the total protein content was strongly influenced by all treatment doses in a concentration-dependent manner, and the highest protein content, 166.53 mg/g FW, was found in treatment T3, compared to the control group at 89.66 mg/g FW, as shown in Figure 9c. The soluble protein content altered to different levels in each treatment group under ZnO-NPs. The soluble protein content reached its maximum (19.08 mg/g FW) at the 40 mg/L ZnO-NP application.

#### 2.1.7. Genome Size Estimation in *E. macrochaetus* under Different Treatments of ZnO-NPs

**Figure 10:**
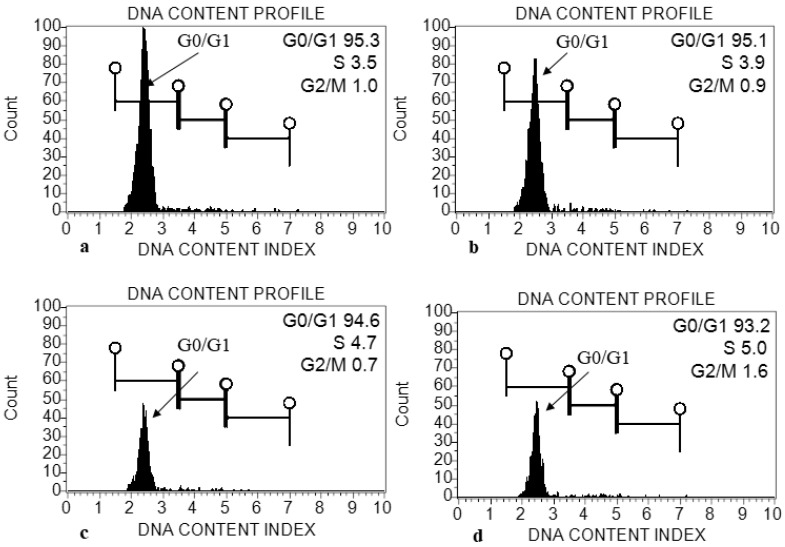
Histogram generated from propidium-iodide-stained nuclei isolated from young leaf of *E. macrochaetus*: (**a**) control (0 mg/L ZnO-NPs); (**b**) treatment T1 (10 mg/L, ZnO-NPs); (**c**) T2 (20 mg/L, ZnO-NPs); (**d**) T3 (40 mg/L, ZnO-NPs).

The genome size (2C DNA content) was estimated in treated plants of *E. macrochaetus*. The leaf tissue was used for the extraction of nuclei using the MB01 buffer. The genome size was estimated from the fluorescence intensity of G0/G1 phase nuclei, which was clearly resolved in histograms (Figure 10). The genome sizes (2C DNA content) were found to be 2.27 pg (10 mg/L ZnO-NPs), 2.25 pg (20 mg/L ZnO-NPs) and 2.24 pg (40 mg/L ZnO-NPs), as compared to control group which had a size of 2.28 pg (Table 1). A minor variation in genome size (2C DNA content) was observed in treated plants as compared to control group.

#### 2.1.8. Estimation of Bioactive Compound Contents in Different Parts of the Plant under Various Treatments of ZnO-NPs

**Figure 11:**
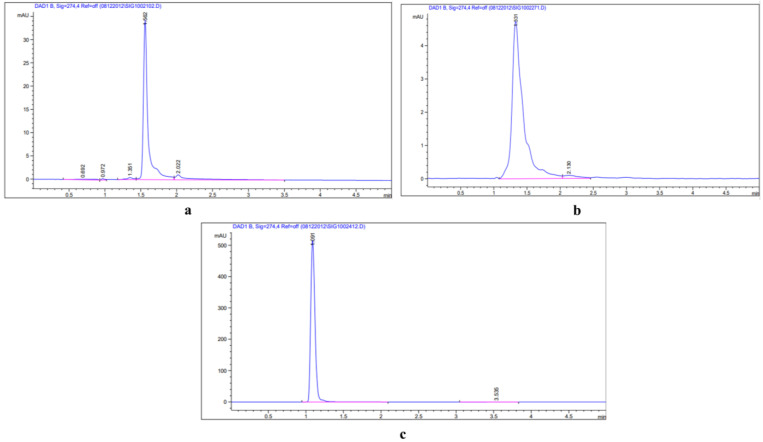
HPLC chromatogram of standard compounds: (**a**) quercetin-3-β-D-glucoside (QBDG); (**b**) luteolin 7-rutinoside; (**c**) p-coumaric acid.

The contents of various bioactive compounds under different treatments of ZnO-NPs are presented in Table 2 and estimated from standard curve obtained from different concentration of standard compounds run in HPLC (Figure 11). The content of quercetin-3-β-D-glucoside (QBDG) (5.59 mg/g DW) was found to be highest in the leaves of treatment T1, and thereafter its content decreased in treatments T2 and T3 as compared to the control group, respectively. All three compounds were varied in different parts of the *E. macrochaetus*, including the leaf, shoot and root. Luteolin 7-rutinoside was highest in treatment T2 (7.53 mg/g DW) compared to control and all other treatments. The content of QBDG decreased in shoots of *E. macrochaetus* under ZnO-NP treatment, and the reduction was significant in treatments T2 (3.32 mg/g DW) and T3 (3.99 mg/g DW) as compared to control (4.55 mg/g DW). However, a non-significant result was found in shoots for the QBDG in treatment T1 as compared to control (Table 1). A similar result was found in the roots, where the content of QBDG was decreased at all concentrations of ZnO-NP applications compared to the control group. All three compounds were recorded higher in leaves, followed by shoots and roots, respectively. The compounds rutinoside and p-coumaric acid were found to be significantly higher in the leaves of treatment T2 (7.53 mg/gDW and 348.55 µg/g DW) compared to the control and all other treatments (Table 1). Furthermore, the content of p-coumaric acid was increased significantly in the roots of treatments T1 (86.67 µg/g DW) and T2 (97.11 µg/g DW) compared to the control group. The content of p-coumaric acid was non-significantly decreased in the roots of treatment T3 (56 µg/g DW) as compared to control (65 µg/g DW).

## 3. Discussion

The problem caused by zinc deficiency in plants is due to the low bioavailability of it in the soil and, consequently, its inadequate content in plant products [34,35,36]. With the increasing application of NPs in the environment and agriculture, an understanding of the ecological and plant growth effect of NPs is of great importance. In edible crops, adverse effects of nanomaterials on the environment and human health have been suggested. In this regard, no laxity in application ought to be tolerated, and dumping issues ought to be considered before commercial use [37]. The coating of nanomaterial on chemicals such as pesticides and fertilizers is an important use of nanotechnology. Plants uptake NPs far more effectively, and in this way, a lower dose is required compared to their natural counterparts. On this basis, NPs appear to be a more potent and less costly alternative and can be expected to promote sustainable large-scale cultivation [20]. ZnO-NPs enable the availability of Zn, which is an important micronutrient for regulating plant development, and ZnO-NPs display low toxicity as compared to other forms of Zn [38,39]. Zinc nanoparticles are approachable, soluble and responsive as compared to conventional Zn fertilizer because of their size at the nanoscale and their more precise surface area [40]. The synthesized biogenic nanoparticles in the present study (<100 nm) confirmed their positive effect on *E. macrochaetus*, as the size of nanoparticles plays an important role from absorption to their transport to the cell.

This study focused on *E. macrochaetus*, which is an important medicinal and endemic plant to Saudi Arabia. The collected seeds of *E. macrochatetus* from the wild of Saudi Arabia were identified, cryopreserved (at low moisture content) and phylogenetically studied using the internal transcribed spacer sequence of nrDNA. Since *E. macrochaetus* was collected from the wild, there were no reports available for its identification using an internal transcribed spacer sequence of nrDNA. The phylogenetic study conducted using the nrDNA-ITS marker is easy and reproducible compared to other molecular markers. The reconstructed phylogenetic tree of *Echinops macrochaetus* along with other *Echinops* species available in the NCBI database (https://www.ncbi.nlm.nih.gov/ (accessed on 16 December 2022)) using an ITS marker showed the highest closeness to *Echinops spinosissimus* (Figure 1). The phylogenetic study based on the ITS marker confirmed its identification and relationship to other species of *Echinops*, which are available in the GenBank database (https://www.ncbi.nlm.nih.gov/ (accessed on 16 December 2022)). DNA barcoding is a novel method for identifying and classifying species based on the nucleotide diversity of conserved sequences. Recently, various studies have shown that DNA markers are highly effective in identifying medicinal plants at genus and species levels. Thus, the ITS marker used in this study showed the phylogenetic relationship of *E. macrochaetus* to the other species of *Echinops*, and a higher capacity of this marker in terms of phylogenetic reconstruction has been proven in other research studies [41,42,43].

Nanoparticles affect plant physiological response in positive as well as negative ways [44,45], and this depends on the concentrations of NPs and plant species used. The objective of the present study was to enhance the biomass and phytochemical compounds in *E. machrochaetus* using biogenic ZnO-NPs. ZnO-NPs are approachable, soluble and responsive as compared to conventional ZnO fertilizer because of their size at the nanoscale and their more specific surface area [40]. The synthesized phytomediated ZnO-NPs using *H. bacciferum* showed a promoting effect on the accumulation of fresh and dry weights of plant parts, including the leaf, shoot and root of *E. macrochaetus*. More biomass accumulation was observed at plants treated with a low concentration of ZnO-NPs (10 mg/L) (Figure 6c). This low concentration of ZnO-NPs showed to be a strong bio-stimulator as compared to other concentrations of ZnO-NPs (20 and 40 mg/L). In some studies, increases in fresh and dry weights as a consequence of ZnO-NP treatment in tomato [30], carrot [46], rice [47] and red perilla [48] were observed. More biomass production in *E. macrochaetus* could be linked to a higher synthesis of chlorophyll and carotenoid contents at a low dose of ZnO-NPs, as measured in treatment T1. Carbon assimilation (biomass accumulation) was increased by both enhanced light (owing to more and longer leaves) and increased chlorophyll content [49]. Remarkably, leaf greenness is typically used as an index of plant health, quality and vigor in the distribution, production and sales method [49]. Thus, the treatments under study upgrade plant value by stimulating leaf coloration. Carotenoid is a non-enzymatic antioxidant, and this way subsidizes the plant redox state [49]. Similarly, the high root to shoot ratio could be correlated to the higher chlorophyll content synthesis in treatment T1. At higher concentrations of ZnO-NPs (20 and 40 mg/L), both chlorophyll and carotenoid contents were decreased in *E. macrochaetus*, which might be due to the reactive oxygen species generation. The toxic effects of ZnO-NPs on *Arabidopsis* are likely caused by reduced chlorophyll contents, which in turn limited photosynthesis in the plants, leading to the reduction in biomass accumulation [50]. The photosynthetic pigment contents were enhanced in the leaves of *Capsicum chinense* [51]. Thus, the improved plant growth of *E. macrochaetus* at different concentrations of ZnO-NPs is due to the enhanced chlorophyll content, since it is a common indicator of the photosynthetic efficiency of a plant, which is one of the most essential factors of its growth [52].

ZnO-NPs showed promoting results on morphometric traits of *E. macrochaetus*, including shoot length, leaf length and leaf number, which were all significantly improved under different treatments as compared to the control group. We chose leaf length to observe the effect of ZnO-NPs, as leaf length is an adequate proxy of individual leaf area in other taxa [53]. Our results are supported by Prasad et al. [54], as their application of ZnO-NPs on peanuts showed significantly increased plant height compared to bulk Zn. Similarly, the application of ZnO-NPs on wheat increased height of the plant [55]. ZnO-NPs (1000 mg/L) positively affected stem diameter, plant height and chlorophyll content and improved fruit yield and biomass accumulation compared to both a control and a ZnSO_4_ treatment [51].

Physiological responses are the first significant changes, which occur when plants interact with different types of elements. Various types of NPs can induce stress responses in plants, affecting morphological traits and physiological, molecular and biochemical reactions in plants [56,57,58]. Additionally, ZnO-NPs have been proposed as a nano-fertilizer to supply Zn to plants. The production of ROS following interactions with NPs has been observed consistently across plant species. In order to mitigate the effects of oxidative stress, plants trigger both enzymatic and non-enzymatic antioxidant defense machinery to scavenge surplus ROS from the cell [59]. Proline, an amino acid, plays an extremely beneficial role in plants exposed to different stress conditions. Lipid peroxidation (as estimated by TBARS content) is indeed triggered by oxidative stress. However, in many plant systems [37] the enhanced activation of the enzymatic antioxidant networks efficiently ameliorates the oxidative damage. The contents of proline and TBARS were increased in the leaves of *E. macrochaetus*, which was observed in all treatments with ZnO-NP application. Our result is confirmed according to Al-Qurainy et al. [28], as their application of ZnO-NPs on tissue-culture-raised shoots of *Ochradenus arabicus* enhanced the levels of both proline and TBARS. Similarly, ZnO-NP treatment on *Momordica charantia* enhanced proline content and up-regulated antioxidant enzyme activity [60].

Abiotic stresses trigger the accumulation of free radicals, which may damage the cell. APX, SOD and GR are key ROS scavenging enzymes, whereas polyphenolics, carotenoids and flavonoids are essential non-enzymatic elements [25]. Among various concentrations of ZnO-NPs applied on *E. macrochaetus*, the enzyme activities of SOD, APX and GR were recorded higher in the plants. Such elevations of enzyme levels in plants under abiotic stress could be correlated to a higher removal of ROS from the cell to reduce oxidative stress. The enzyme SOD catalyzes the detoxification of O^−2^ into either O_2_ or H_2_O_2_, and ascorbate peroxidase (APX) detoxifies peroxides such as H_2_O_2_ using ascorbic acid as a substrate; both of these were up-regulated in plants that underwent NP treatment [61]. Hussain et al. [26] performed an experiment on wheat with ZnO-NPs (100 ppm), and the enhanced antioxidant enzyme activities decreased the oxidative stress. Various other studies have shown that ZnO-NPs can induce oxidative stress and modify the activity of non-enzymatic and enzymatic compounds [62,63], which function together to defend plant cells against oxidative stress. ZnO-NP treatment boosted the antioxidant activity and level of total anthocyanins in red *Perilla frutescens* [48].

Heavy metals, both essential and non-essential, yield symptoms of toxicity in plants when used in high concentrations. Some heavy metals play important roles in the functioning of plants as key components of photosystems and enzymes. Many studies revealed that metal nanoparticles may act as elicitors that stimulate the production of secondary metabolites in plants [64] (review). Zinc insufficiency affects the metabolism of carbohydrates, and a high concentration of zinc is known to affect flavonoid metabolism [65]. Our study revealed the promoting effect of ZnO-NPs on bioactive compound accumulation in different parts of *E. macrochaetus*. All studied compounds (quercetin-3-β-D-glucoside, luteolin 7-rutinoside and p-coumaric acid) were detected in various parts of *E. macrochaetus*, including the leaf, root and shoot, at different treatment concentrations of ZnO-NPs. Variation was observed in the contents of compounds in different parts of *E. macrochaetus* under ZnO-NP treatments. The result of Borovaya et al. [66] revealed rutin biosynthesis enhancement in a hybrid of buckwheat after exposure to zinc at a high concentration of 1010–1212 mg/L. The result of Karimi et al. [67] showed that low concentrations of NPs have been found to promote plant growth and the production of secondary metabolites. In another study, ZnO-NP treatment enhanced the polyphenolic content in plants [28,48,60]. Similarly, the polyphenolic compounds (lignans and neolignans) were enhanced in cell suspension cultures of *Linum usitatissimum* under ZnO-NP treatments [29].

The most pronounced effect was observed on *E. macrochaetus* at a low concentration of ZnO-NPs, which enhanced the content of these polyphenolic compounds in leaves and shoots. The content of p-coumaric was enhanced in *Hypoxis hemerocallidea* in plants grown on standard Murashige and Skoog (MS) medium containing the normal 30 µM Zn concentration [68]. Excess concentrations of nanoparticles produce reactive oxygen species (ROS), which can cause damage to the basic building blocks of the cell, including proteins, DNA and lipids. In response to ROS, cells produce various types of secondary metabolites to remove ROS. Variations in the accumulation of secondary metabolites in different parts of *E. macrochaetus* might be due to the generation of ROS at different doses of ZnO-NPs. These reactive oxygen species (ROS) themselves act as signaling molecules, which are capable of inducing plant secondary metabolism [69]. Various concentrations of ZnO-NPs produced different effects on the accumulation of bioactive compounds in various organs of *E. macrochaetus*. No part of *E. macrochaetus*, including the leaf, shoot and root, showed a similar trend in the accumulation of bioactive compounds, including QBDG, luteolin 7-rutinoside and p-coumaric acid. However, the mechanism of production of secondary metabolites in plants under nanoparticle application is still poorly understood.

Many studies with NP application have shown a certain degree of phytotoxicity, mainly at high concentrations [70]. These NPs, directly or indirectly via the induction of oxidative stress and production of reactive oxygen species (ROS), often disturb the physical or chemical structures of DNA and induce both genotoxic and cytotoxic stresses. Such a circumstance, in turn, leads to genome instability, and thus ultimately affects plant growth and yield. In the present study, different concentrations of biogenic ZnO-NPs were applied to *E. macrochaetus* plants to enhance their biomass and polyphenolic compounds, as Zn participates in many biological activities. Different doses of ZnO-NPs positively affected the physiology of *E. macrochaetus* as well as morphological traits, rather than producing negative effects. High metal concentrations in the cytoplasm and nucleus could certainly induce genotoxicity and cytotoxicity to plants. Therefore, genome size (2C DNA content) was estimated in *E. macrochaetus* under different treatment doses of ZnO-NPs using flow cytometry, which is easy to perform. The histogram generated with flow cytometry (FCM) using the MB01 buffer [71] from all treated plants (ZnO-NPs) showed a minor variation in genome size compared to the control group. The variations in plant genome size are caused by lineage-specific molecular mechanisms in both DNA removal and DNA amplification that modify the total amount of nuclear DNA content [72]. However, the minor variation in the genome size of *E. macrochaetus* might be due to the generation of genotoxicity. The mitotic index in terms of 4C/2C indicated the genotoxicity in *E. macrochaetus* under ZnO-NP treatment. Genotoxicity caused by ZnO-NPs was assessed in different plant species using different molecular approaches and the mitotic index [73], which confirmed the results obtained on *E. macrochaetus*.

## 4. Materials and Methods

### 4.1. Plant Collection and Identification

The seeds of *Echinops macrochaetus* and *Heliotropium bacciferum* leaves were collected from the Abha and Al-Heir regions of Saudi Arabia, respectively. The plant was identified by a taxonomist at the Department of Botany and Microbiology, King Saud University, Saudi Arabia. Some seeds of *E. macrochaetus* were used for the present study and the remaining seeds were cryopreserved after the removal of moisture content.

### 4.2. Isolation of Genomic DNA, PCR and Sequencing

The genomic DNA was extracted using the modified CTAB method [74]. The nrDNA-ITS locus was amplified using the forward (ITS4: 5′-GTCCACTGAACCTTATCATTTAG-3′) and reverse primers (ITS1: 5′-TCCTCCGCTTATTGATATGC-3′). These universal primers were synthesized from Macrogen Company, Geumcheon-gu, Seoul, South Korea. The reaction mixture consisted of 21 µL distilled water added in PuReTaq^TM^ PCR beads (GE Healthcare, Great Britian, Little Chalfont, Amersham place, UK), 1 µL (25 ng) genomic DNA, 1.5 µL 20 pM primer (each primer) and remaining water. The PCR reaction was run in replicate for reproducibility of the results. The reaction was performed in a Veriti 96 well thermalcycler (Thermo Fisher Scientific, Jln Kilang Timor, Singapore) with the following settings: first cycle at 94 °C for 4 min, 30 cycles at 94 °C for 1 min, annealing at 50 °C for 1 min, extension at 72 °C for 1 min, and a final extension at 72 °C for 4 min. The PCR products were sequenced at Macrogen, Geumcheon-gu, Seoul (South Korea).

### 4.3. Aqueous Leaf Extract Preparation for Synthesis of ZnO-NPs

The mature leaves of *Heliotropium bacciferum* were used for natural extract preparation. The leaves were air-dried and ground to make fine powder. For preparation of leaf extract, the powder was added into 120 mL milli Q water (250 mL conical flask) and kept in a water bath at 100 °C for 25 min. The whole mixture was taken out from the water bath and filtered through Whatman filter paper No. 1.

### 4.4. Natural-Extract-Mediated ZnO-NP Synthesis

ZnO-NPs were prepared according to the method as followed by Al-Qurainy et al. [28] with minor modifications. An amount of 75 mL of freshly prepared *Heliotropium bacciferum* leaf extract was added into a 0.05 M Zn(NO_3_)_2_ solution at 60 °C and kept at constant stirring overnight. Upon the addition of 0.1 N NaOH drop by drop in the above reaction mixture, a brown whitish color appeared as in the synthesis of nanoparticles. The reaction mixture was cooled down and centrifuged at 9000 rpm at 4 °C for 15 min to make a pellet. The pellet was washed three times with double-distilled water followed by alcohol to remove impurities. The calcination was carried out in a furnace (DKN 602, Yamato Scientific Co., Ltd., Tokyo, Japan) at 500 °C for 4 h.

### 4.5. ZnO-NP Characterization

Plant-mediated synthesized ZnO-NPs were observed as the precipitation of brown whitish particulates, which was confirmed by ultraviolet–visible (UV–Vis) spectroscopy. All analyses related to characterization were carried out at King Saud University, Riyadh, Saudi Arabia. Particle stability, size and shape were analyzed using the zeta potential and transmission electron microscopy (TEM) techniques. Fourier transform infrared spectroscopy (FT-IR) was performed to analyze the different functional groups present in the synthesized nanoparticles.

### 4.6. Seed Sterilization and Pot Experiment

The collected mature seeds of *E. macrochaetus* were washed with tap water for 25 min followed by double-distilled water to remove dust and microbes. The seeds were treated with 50% bleach for 5 min for surface cleaning and subsequently the seeds were washed for 5 min in sterile double-distilled water. After sterilization, percent germination was tested. The seeds were sowed in pots (size: 12 cm × 10 cm) containing 1 kg of soil (peat moss and pearlite-3:1). *E. macrochaetus* plants were raised in a growth chamber with the following conditions for their proper growth: 16 h/8 h, 25 ± 1 °C day/night cycle.

### 4.7. Treatment of Plant with ZnO-NPs

The plants were treated with various concentrations of ZnO-NPs (T1 = 10 mg/L, T2 = 20 mg/L and T3 = 40 mg/L) after 30 days of sowing when plants were at the four-leaf stage. Plants were irrigated one time per month with ZnO-NPs and another time with double- distilled water every after fifteen days. The control plants were grown without nanoparticles (0.0 mg/L ZnO-NPs) and were treated with double-distilled water (DDW) at the same time intervals. The sampling was performed after 120 days of sowing.

### 4.8. Morpho-Physiochemical Analysis

#### 4.8.1. Biomass Determination

The biomass was measured after 120 days of sowing the seeds. Fresh and dry weights of *E. macrochaetus* were measured at different treatments of ZnO-NPs. The aboveground parts (shoots with leaves) were cut and weighed to measure the fresh and dry weights, then dried at 65 °C for 48 h to measure the dry weight. Analysis was carried out in replicates (*n* = 3) for each combination of treatments.

#### 4.8.2. Morphological Traits

The shoot length, leaf number and leaf length were measured, and comparison was performed with the control group and among treated plants.

#### 4.8.3. Determination of Total Chlorophyll and Carotenoids

The method developed by Lichtenthaler [75] was followed for estimation of total chlorophyll and carotenoids. Fresh leaves (0.1 g) were washed in distilled water and chopped in small pieces. The chlorophyll was extracted in 100% acetone. The incubation was performed at room temperature overnight and thereafter centrifuged at 500× *g* for 5 min. The absorbance was taken at 663 nm and 645 nm using UV–vis spectrophotometer:

Chlorophyll a: Ca = 12.25 A663.2 − 2.79 A646.8 (µg per ml solution).
Chlorophyll b: Cb = 21.50 A646.8 − 5.10 A663.2 (µg per ml solution).
Total carotenoids: Cx + c = (1000 A470 − 1.82 Ca − 85.02 Cb)/198 (µg per ml solution).

The calculated chlorophyll and carotenoid contents were expressed in μg/g FW.

#### 4.8.4. Proline Estimation

Fresh leaves (0.2 g) were used for the estimation of proline using the method established by Hanson et al. [76]. The fresh samples were ground in 5 mL, 3% aqueous sulphosalicylic acid. The samples were centrifuged at 9000× *g* for 10 min and supernatants (2 mL) were taken in another tube. An amount of 2 mL of each (acid ninhydrin and acetic acid) was added in the above mixture. The samples were incubated in boiling water for 1 h, and the reaction was terminated by putting the samples in an ice bath. Toluene (4 mL) was added in the above samples and they were vortexed. The aqueous phase was separated from chromatophore-containing toluene. The proline content was estimated in samples by taking absorbance of chromatophore containing toluene at 520 nm (Model UB-1800, Shimadzu, Japan). The unit of proline content was expressed in μg/g FW.

#### 4.8.5. Thiobarbituric Acid Reactive Substances (TBARS)

TBARS content was estimated in fresh leaves using the method of Cakmak and Horst [77]. The leaf samples (0.25 g) were homogenized in 10 mL trichloroacetic acid (TCA; 0.1%, *w*/*v*). The supernatant was collected at 12,000× *g* for TBARS estimation. An amount of 4 mL of 0.5% (*w*/*v*) TBA in 20% (*w*/*v*) TCA was added in 2 mL of supernatant taken from the above step and placed for 30 min at 90 °C in a water bath. TBARS content was measured from the absorbance taken at 532 and 600 nm wavelengths using a spectrophotometer.

$$\mathrm{TBARS}\;\left(\mathrm{nmolg}-1fw\right)=\frac{\left(A532-A600\right)\times V\times 100}{155\times \mathrm{extinction}\;\mathrm{coefficient}\times w\times 1}$$

A532 represents absorbance at 532 nm, A600 represents absorbance at 600 nm, V = extraction volume and w= fresh weight of tissue.

#### 4.8.6. Total Crude and Soluble Protein Estimation

The harvested leaf samples were directly frozen in liquid nitrogen and stored at −80 °C until protein extraction. Total protein was extracted using phosphate buffer containing 1% of PVP, Triton X100 and β-mercaptoethanol. Fresh leaves (0.2 g) were homogenized in 1 mL extraction buffer (0.1 M phosphate buffer, pH 7.2). The crude homogenate was centrifuged at 5000× *g* for 10 min. The supernatant was taken in another tube, and the total crude protein was measured at 280 nm using the Nanodrop 8000 spectrophotometer.

The total soluble protein was extracted using the method as described by Singh et al. [78] with minor modifications. The fresh plant leaves (0.2 g) were powdered in a pestle and mortar with liquid nitrogen. The powdered samples were homogenized using phosphate buffer (0.1 M, pH 7.5). The supernatant was transferred to a fresh tube containing 2 mL of TCA (10%) in acetone with β-mercaptoethanol (0.07%) for protein precipitation. The protein pellet was obtained after centrifugation at 13,000 rpm at 4 °C for 15 min. The pellet was washed thrice with chilled acetone supplemented with β-mercaptoethanol (0.07%) and EDTA (2 mM). Final washing was performed using pure acetone. The pellet was dissolved in rehydration buffer and measured at 280 nm using the Nanodrop 8000 spectrophotometer.

#### 4.8.7. Superoxide Dismutase (EC 1.15.1.1)

The activity of superoxide dismutase (SOD) was measured using the method as developed by Dhindsa et al. [79]. The sample absorbance was recorded at 560 nm along with a blank using the UV–vis spectrophotometer (Model UB-1800, Shimadzu, Japan). A 50% decline in color was considered as one enzyme unit (EU) mg^−1^ protein min^−1^.

#### 4.8.8. Ascorbate Peroxidase (EC 1.11.1.11)

The ascorbate peroxidase (APX) activity was checked using the method of Nakano and Asada [80]. Fresh leaves (0.2 g) were ground in 1 mL of extraction buffer (50 mM phosphate buffer), 1% Triton X100 (*w*/*v*), 1% PVP (*w*/*v*) and 0.3 mM EDTA. The supernatant was taken after centrifugation to analyze enzyme activity at 290 nm using the UV–vis spectrophotometer. The extinction coefficient (e) 2.8 mM^−1^ cm^−1^ was used for calculation of APX activity and enzyme units (EU) expressed as mg^−1^ protein min^−1^.

#### 4.8.9. Glutathione Reductase (EC 1.6.4.2)

The glutathione reductase (GR) activity was measured in fresh leaves at 340 nm wavelength followed by the protocol of Rao [81]. The GR activity was calculated using the molar absorptivity constant of NADPH (6.2 mM^−1^cm ^−1^) and expressed as EU mg^−1^ protein min^−1^.

### 4.9. Assessment of Genome Size (2C DNA Content)

The nuclei were extracted using the protocol (MB01) as developed by Sadhu et al. [71]. The extraction buffer consisted of Triton X-100, 25 mM Na_2_EDTA, 20 mM MOPS, 0.7 mM spermine, 20 mM NaCl, 80 mM KCl, 1% (*w*/*v*) PVP, 0.5% (*v*/*v*) β-mercaptoethanol and 0.2% (*v*/*v*); pH 7.4.

#### 4.9.1. Nuclei Extraction

A small amount of fresh young leaves (25 mg) was used from treated and untreated plants of *E. macrochaetus* for the extraction of nuclei. The leaf was sliced with a sharp blade into 0.5–0.6 mm size in cold nuclei buffer [71]. The nuclei were filtered through double nylon mesh (pore size-20 µm, procured from Macrokun (Shijiazhuang, China)). The suspension of filtered nuclei was made to 700 µL. The nuclei were stained with 50 µg/mL PI dye (propidium iodide) and kept at 4 °C for 10 min. The isolation of nuclei from a standard plant (*Solanum lycopersicum*) as provided by Dr. Jaroslav Dolezel was performed independently with the same buffer to locate its peak position. Relative 2C nuclear DNA content (genome size) of *E. macrochaetus* was calculated by comparing with the genome size of the standard plant.

#### 4.9.2. Flow Cytometry Analysis

Nuclear DNA content (2C DNA content) of *E*. *macrochaetus* was calculated according to Doležel et al. [82]. *Solanum lycopersicum* (2C = 1.96 pg) was used for estimation of genome size of *E. macrochaetus*. The acquisition was stopped at 5000 propidium-iodide-stained nuclei in flow cytometer FACS Muse cell analyzer (Sigma, St. Louis, MO, USA). The histograms generated in Muse cell analyzer were used for estimation of genome size. The genome size (2C DNA content) of *E. macrochaetus* was calculated according to the formula

$$2C\;\mathrm{DNA}\;\mathrm{content}\;\left(\mathrm{genome}\;\mathrm{size}\right)=\frac{\mathrm{Florescence}\;\mathrm{mean}\;\mathrm{intensity}\;\mathrm{of}\;E.\;macrochaetus}{\mathrm{Floresence}\;\mathrm{mean}\;\mathrm{intensity}\;\mathrm{of}\;\mathrm{standard}}\times \mathrm{2C DNA content of standard}$$

The mean and standard deviations were calculated for 2C values of each treatment based on replicates.

### 4.10. Extraction and Estimation of Bioactive Compounds

Plant parts, including leaves, roots and shoots, were harvested from treated and untreated plants. The samples were dried in an oven for removal of moisture content. Then, a 0.2 g powder of all plant parts was added in 25 mL methanol for the extraction of bioactive compounds. The mixture was kept for 12 h on a rotatory shaker, and it was filtered through Whatman filter paper No. 1. The rotary vacuum evaporator was used to concentrate the filtrate under reduced pressure at 40 °C. Finally, the obtained extract was dissolved in methanol for HPLC analysis.

The bioactive compound quantification was accompanied with an HPLC system with a UV–vis diode array detector (DAD). The extracts of different parts of *E. macrochaetus* were analyzed using a Waters System (Agilent Technologies 1290 Infinity system) attached to a diode array detector with a 200–400 nm detection range. The separation of compounds in crude extracts was performed in a ZOBRAX RX-C18 column (4.6 × 150 mm), in which the mobile phases were at flow rates of 1 mL/min (luteolin 7-rutinoside), 0.80 mL/ min (quercetin 3-β-D-glucoside; QBDG) and p-coumaric acid (1 mL/min) with a run time of 5 min and injection volume of 1 µL. The standard solutions of quercetin-3-β-D-glucoside, luteolin 7-rutinoside and p-coumaric acid were used for the estimation of compounds in the sample extract. The mobile phase methanol and acetonitrile were set up in the ratio of 65:35 for QBDG, for luteolin 7-rutinoside (methanol and 0.6% acetic acid (*v*/*v*) in water (65:35), and for p-coumaric acid (acetonitrile: water, (40:60; *v*/*v*)). All extracted compounds in the crude extract, including QBDG, luteolin 7-rutinoside and p-coumaric acid, were estimated at a wavelength (λ) = 274 nm. The retention times for standard compounds were 1.54 min, 1.34 min and 1.1 min for QBDG, luteolin 7-rutinoside and p-coumaric acid, respectively. The luteolin 7-rutinoside, QBDG and p-coumaric acid were calculated using the linear regression equations obtained from the standard curve: Y = 666.7X − 4.89 (R^2^ = 0.999, Y: peak area, X: rutin content); Y= 1449.1X + 21.105 (R^2^ = 0.996, Y= peak area, X= QBDG content) and Y = 20681X − 12.13 (R^2^ = 0.999, Y = peak area, X= p-coumaric acid content), respectively.

### 4.11. Statistical Analysis

All sample analyses were executed in three replicates, and the final means are presented with their standard deviations (SDs).The experiment was repeated twice and conducted in controlled conditions. One-way ANOVA using SPSS version 16 (IBM, SPSS Statistics 26) was performed for data analysis of different studied parameters. The significance of differences among means was ascribed using Duncan test at 5% level of significance.

## 5. Conclusions

This is the first study regarding the effect of the phytomediating role of ZnO-NPs on *Echinops macrochaetus* for its growth improvement and polyphenolic compound accumulation. The morphometric and biochemical parameters results revealed the positive effect of ZnO-NPs on leaves, shoots and roots in terms of plant biomass and bioactive compound accumulation. Low concentrations of ZnO-NPs (10 mg/L) showed more positive effects on the whole plant than the high concentrations. A minor variation in genome size was found in all treated plants as compared to untreated plants, which indicated the less toxic effects of the applied doses of ZnO-NPs on *E. macrochaetus*. Moreover, the genome size, estimated for the first time in *E. macrochaetus*, will help to the researchers in whole-genome sequencing and other molecular biology studies. Thus, the applied doses of ZnO-NPs on *E. macrochaetus* were ascertained favorable as revealed from the results of various studied parameters; however, more analyses should be conducted on this endemic species to describe the differences in polyphenolic compound accumulation and morphological trait variations under phytomediated ZnO-NP treatment. Thus, the present research showed that biogenic nanoparticles (ZnO-NPs) could be used as elicitors to improve the biosynthesis of bioactive compounds in various medicinal plants to enhance biological activity.

## Figures and Tables

**Table 1:** Genome size (2C and 4C DNA content) and mitotic index calculated from generated histogram using flowcytometry.

Treatment	DNA Content (pg) per 2C (G0/G1) (Mean ± SD)	DNA Content (pg) per 4C (S Phase) (Mean ± SD)	Mitotic Index (4C/2C)
Control (0 mg/L, ZnO-NPs)	2.2813 ± 0.01 a	4.01 ± 0.08 b	1.761
T1 (10 mg/L, ZnO-NPs)	2.275778 ± 0.03 a	3.88 ± 0.08 ab	1.708
T2 (20 mg/L, ZnO-NPs)	2.257111 ± 0.05 a	3.84 ± 0.10 a	1.695
T3 (40 mg/L, ZnO-NPs)	2.249022 ± 0.04 a	3.7 ± 0.08 a	1.681

Data are means of three replicates (mean ± SD). Different letters represent significant value (*p* < 0.05).

**Table 2:** Content of bioactive compounds in leaves, shoots and roots of *E. macrochaetus*.

Treatment	Quercetin-3-β-D-Glucoside (mg/g DW)	Luteolin 7-Rutinoside (mg/g DW)	p-Coumaric Acid (µg/g DW)
Leaf			
Control	4.52 ± 0.11 b	5.99 ± 0.27 c	270.22 ± 7.91 c
T1	5.59 ± 0.23 a	7.01 ± 0.14 b	313.16 ± 8.94 b
T2	5.31 ± 0.04 a	7.53 ± 0.39 a	348.55 ± 2.13 a
T3	4.09 ± 0.13 c	5.63 ± 0.25 d	216.64 ± 9.90 d
Shoot			
Control	4.55 ± 0.16 a	4.94 ± 0.27 c	200.59 ± 9.73 b
T1	4.27 ± 0.17 a	6.01 ± 0.12 b	277.96 ± 11.91 a
T2	3.32 ± 0.08 c	6.50 ± 0.10 a	263.45 ± 6.43 a
T3	3.99 ± 0.08 b	4.63 ± 0.25 d	176.80 ± 7.56 c
Root			
Control	0.88 ± 0.03 a	1.52 ± 0.11 ab	65.20 ± 5.91 b
T1	0.69 ± 0.07 b	1.40 ± 0.06 ab	86.67 ± 7.17 a
T2	0.58 ± 0.04 c	1.46 ± 0.24 a	97.11 ± 9.85 a
T3	0.31 ± 0.05 d	1.27 ± 0.09 c	56.50 ± 6.77 b

Data are means of three replicates (mean ± SD). Different letters represent significant values (*p* < 0.05).

## Data Availability

All the data supporting the findings of the current study are available in this article.
